# Family planning knowledge, experiences and reproductive desires among women who had experienced a poor obstetric outcome in Lilongwe Malawi: a qualitative study

**DOI:** 10.1186/s40834-018-0075-8

**Published:** 2018-10-17

**Authors:** Agatha Bula, Dawn M Kopp, Suzanne Maman, Lameck Chinula, Mercy Tsidya, Jennifer H Tang

**Affiliations:** 1 UNC Project-Malawi, Private Bag A-104, Lilongwe, Malawi; 2 UNC Department of Obstetrics & Gynecology, Chapel Hill, NC USA; 3 Kamuzu Central Hospital, Lilongwe, Malawi; 4 UNC Department of Health Behavior, Chapel Hill, NC USA; 5 Malawi College of Medicine Department of Obstetrics & Gynaecology, Blantyre, Malawi

**Keywords:** Family planning, Stillbirth, Neonatal death, Poor obstetric outcome, Reproductive desire, Misconceptions, Malawi

## Abstract

**Background:**

Perinatal mortality is unacceptably high in low-income countries, including Malawi. Use of family planning to encourage birth spacing may optimize outcomes for subsequent pregnancies. However, the reproductive desires and family planning knowledge of women who have experienced a stillbirth or neonatal death in resource-poor settings are not well understood.

**Methods:**

We examined family planning knowledge, contraceptive practices and barrier to contraceptive use among women who had experienced a poor obstetric outcome at Bwaila Hospital in Lilongwe, Malawi. We performed individual in-depth interviews or through focus group discussion with women who had experienced a stillbirth or early neonatal death, 4–8 weeks after their delivery. NVivo software was used to analyze data for recurrent patterns and themes, and central ideas were extracted to identify the data’s core meanings.

**Results:**

We interviewed 46 women who had experienced a poor obstetric outcome. Overall, women were aware of both modern and traditional family planning methods, and the majority were in favour of modern versus traditional methods. They also had knowledge about risks for future complications if they have a short inter-pregnancy interval. However, they faced conflict about whether to use family planning methods for their health, as suggested by their relatives and friends, or to have another child to fulfil their husband’s desire, especially among those with no living child. Some had fear about side effects, while others were concerned that use of family planning methods without involving the husband could bring misunderstandings within the family. A number of women had misconceptions about family planning methods, which also served as a barrier to their use.

**Conclusion:**

Although women with a poor obstetric outcome are aware of modern family planning and its health benefits after their delivery, their decision to use a method is complicated by their own desire to protect their own health and the husband’s desire for a child, particularly among those women with no living children coupled with fear of side effects and misconceptions. These findings suggest the importance of counselling both the affected woman and her husband about the benefits of family planning use, even after a poor obstetric outcome, to jointly choose the method they feel comfortable to use and dispel any misconceptions.

**Trial registration:**

Clinicaltrials.gov NCT02674542.

## Background

Perinatal deaths account for 40% of infant mortality globally, resulting in about 3 million stillbirths and 4 million neonatal deaths yearly. The burden of perinatal mortality rate is unacceptably high in low-income countries, especially among those in sub-Saharan Africa and south central Asia, with an average perinatal mortality rate of 50 deaths per 1000 live births, compared with 10 deaths per 1000 live births in high-income countries [1, 2]. In a systematic review to determine the timing of overall and cause-specific neonatal deaths in developing country settings, the pooled results showed that about 62% of the total neonatal deaths occurred during the first 3 days of life, and two thirds of these deaths occurred during the first day alone [3].

Progress in reducing neonatal mortality is being made in all regions; however, sub-Saharan Africa has seen one of the slowest reductions globally (1.5% per year from 2000 to 2010), and maternal and neonatal mortality remain major public health challenges [4]. In Malawi, perinatal mortality remains a major public health problem. Recent estimates show a perinatal mortality ratio of 35 deaths per 1000 live births. Perinatal mortality is highest among women in which the previous pregnancy interval was less than 15 months (55 deaths per 1000 live births), followed by women with a first pregnancy (48 deaths per 1000 live births) [5].

Family planning has been shown to reduce maternal and infant mortality [4, 6, 7]. However, contraceptive use in many resource-limited countries, including Malawi, remains low. In 2015, 64%t of married or in-union women of reproductive age worldwide were using contraception. However, contraceptive use was much lower in the least developed countries (40%) and was particularly low in Africa (33%) [8]. Similarly, in Malawi high and persistent fertility rates have been seen for a long period of time, partly due to low levels of contraceptive use among women. The 2015–2016 Malawi Demographic Health Survey (MDHS) revealed that contraceptive use increased with the number of living children: 58% among those with 1–2 children, compared with 4% among those with no living child [5].

Contraceptive practices and intentions among women with a recent perinatal death have not been explored. Therefore, we examined family planning use and experiences of postpartum women who had a recent stillbirth or early neonatal death in Malawi to address this gap in the literature. Understanding the knowledge, attitudes, and practices for family planning use among women who experienced a poor obstetric outcome is crucial to increase their contraceptive use and reduce poor obstetric outcomes in the future.

## Methods

This qualitative study employed both individual in-depth semi-structured interviews (IDIs) and focus groups discussions (FGDs). Before carrying out this study, ethical approval for the conduct of this study was obtained both from the National Health Sciences Research Committee of Malawi (Protocol #1354) and the University of North Carolina School of Medicine Institutional Review Board (#14-2677). All study participants provided written informed consent at the time of enrollment in the local language (Chichewa) spoken by the majority of people in the district.

### Study setting and population

The study participants were recruited from Bwaila Hospital, a district government hospital in Lilongwe, the capital city of Malawi. Bwaila has approximately 15,000 deliveries annually, of which 2900 are preterm. Between 80 and 110 cases of birth asphyxia (a portion of which result in a stillbirth or neonatal death) occur each month on the labor ward. The inclusion criteria for the study were: 1) current admission to the postpartum ward at BwailaHospital; 2) delivery of a stillborn fetus over 28 weeks gestation or with a birthweight >1000 g, or delivery of a live born infant weighing >1000 g with a neonatal demise in the first 7 days of life; 3) ability to speak Chichewa (the local language) or English fluently; and 4) age 18–45 years old.

### Recruitment and data collection

To ensure our ability to reach thematic saturation, we decided to enroll 60 participants to account for an approximated loss-to-follow-up of up to 20%, given the 4–8 week delay in completing the interviews. This number was predetermined for our submission to the IRB. We enrolled women with and without living children from prior pregnancies from the postnatal wards at a 1:1 ratio. A demographic form was completed for women who consented to enrol in the study. This form collected information about age, number of other living children, HIV status, marital status, completed education, and occupation. Since HIV testing is performed on all Malawian women during antenatal care unless they opt out, HIV status for women who were enrolled in this study was determined by verifying the participant’s health passport (a government-issued personal medical record booklet kept by the patient) with the participant’s permission at the time of enrollment.

Enrolled women were traced 4–8 weeks later to either participate in an in-depth interview (IDI) or a focus group discussion (FGD). All IDIs and FGDs were conducted by an experienced bilingual researcher (M.T.). Based on prior similar studies, we felt that thematic saturation could be achieved within twenty IDIs and four FGDs [9–11]. The IDIs took place in the participant’s home or another private setting depending on the woman’s choice, and the FGDs (of 6–8 participants each) took place in a private conference room on the campus of Kamuzu Central Hospital in Lilongwe, Malawi. Similar structured interview guides were used during IDIs and FGDs. However, a specific aim of the FGDs was to facilitate brainstorming about potential birth spacing interventions, whereas the IDIs focused more on individual and social influences on birth spacing that may be too personal to share in a group setting. During the FGDs, another member of the research team took notes. Summary notes were written immediately after the FGD and IDI to document key takeaways. The research team reviewed the summary notes and changes were made to the guides on areas that required further probing.

### Data analysis

Interviews and focus group discussions were audiotaped with the participants’ permission, transcribed and translated into English. All transcriptions and translations were completed simultaneously with data collection by the same researcher (M.T.). The accuracy of the translations of the IDIs and FGDs and summary notes was verified by two other bilingual members of the research team (A.B. and G.H as they were being conducted, transcribed and translated, and feedback was given to the interviewer on areas that needed more probing to identify emerging themes. The interview guides were also revised as needed to further evaluate new themes that emerged during the review of the transcripts, which was done in tandem with study enrolment.

A codebook was developed via an iterative process based on identified domains, themes, and sub-themes identified during initial transcript review. The team met periodically to discuss the codebook as it was being developed and reached a consensus on the final version of the codebook after all coding was completed Inductive codes emerging from the data during the analysis process were also discussed and added to the codebook. This codebook was assigned to sections of the text by two researchers (D.K and A.B) independently using the qualitative software NVivo® 10 with cross-coding used to ensure consistency between the two analysts. Recurrent themes were identified based on these initial codes, and we wrote memo summaries of the main findings. Any discrepancies were resolved through discussion. Matrices and tables were used to organize the data and display these to facilitate analysis that integrated both IDIS and FGDs based on the conceptual domains and themes. After completion of coding, the research team identified representative statements to help better elucidate the identified themes.

This manuscript focuses on the five family planning-related domains that were discussed during the IDIs and FGDs: 1) Knowledge about family planning methods (both modern and traditional), 2) Conflict in decision-making for family planning use following a bad obstetric outcome, 3) Attitudes towards family planning use after a bad obstetric outcome, 4) Family planning practices of participants following a poor obstetric outcome, and 5) Barriers to modern family planning method use following a poor obstetric outcome.

## Results

### Characteristics of women

In total, 46 (76.7%) of the 60 enrolled women participated in FGDs or IDIs; the remaining 14 enrolled women either could not be traced or declined continued participation in the study at the time that they were traced. Most women were between 18 and 34 years (87%), and the majority were married (Table 1). Although 30 women with living children and 30 women without living children were enrolled, slightly more women who participated in the study (*n* = 28, 61%) had living children. Those with living children had a median of two children. Seven women (15%) were HIV-infected. All women interviewed were of low socio-economic status, and the majority had no education and hence did not have any formal employment.

**Table 1:** Demographic characteristics of study participants

Characteristic	Frequency (*n* = 46)	Total percentage
Age		
18–24 years	24	52
25–34 years	16	35
35 years ≥	6	13
Marital status		
Married	39	85
Not married	7	15
HIV status		
HIV uninfected	39	85
HIV infected	7	15
Education		
None	12	26
Some primary	21	46
Secondary or more	13	28
Pregnancy outcome		
Stillbirth	23	50
Neonatal death	23	50
Living children		
Living children	28	60.9
No living children	18	39.1

#### General knowledge about family planning methods

The majority of participants demonstrated high levels of awareness about modern family planning methods and were able to name some of the most common contraceptive methods available, including the Depo Medroxyprogesterone Acetate (DMPA) injectable, the copper Intrauterine Device (IUD), oral contraceptives, implants, vasectomy, tubal ligation and male condoms:

*Interviewer: “What family planning methods have you heard about?”*

*Respondent: “I have heard about loop (meaning intrauterine device), injection which others call it Depo, Norplant and natural family planning methods where a woman does not use any drugs or hormones.” (FGD 004)*

Nearly all women interviewed in this study reported to have attended antenatal visits, where education sessions about pregnancy, childbirth, family planning and child care are given to all women. As a result, a majority mentioned antenatal and under-five clinics (pediatric clinics for children who are less than 5 years of age) as major sources of information for modern family planning methods. One respondent noted that she had been sensitized about modern contraceptives at the clinic through local songs and drama:

*“I heard about it from the under-five clinic. Service providers were teaching us songs with family planning messages. {Respondent singing}: Why should I have all these children that are supposed to be supported by me? I should not look old while I am still young. So I learnt something from the songs.” (IDI 035)*

Notably, a considerable number of women mentioned their friends and family members with previous experience of contraceptive use as a source of information for modern family planning methods, which demonstrated that many people in their communities are in favour of family planning use. These friends mainly played a role in sensitizing the women about family planning and encouraging them to go to the hospital to receive first-hand information from service providers:

*“When I asked one of my friends about what happens, she told me to go to the clinic myself so that they (service providers) can tell me the whole process of permanent contraception, and then I can decide whether to do it. So when I go to the clinic next week I will ask them how it happens.” (IDI 035)*

On the other hand, some women also mentioned natural family planning methods, especially withdrawal. However, despite having the knowledge about withdraw methods they admitted to lack knowledge on how it works. Catholic hospitals were mentioned by the majority of women as the most common source of information for these methods:

*Interviewer: “What happens with natural family planning methods?”*

*Respondent: “Families were mobilized to go for education on natural methods at Likuni Hospital {a Catholic mission hospital within Lilongwe District}, but I did not go, and I don’t know what happens, but someone just told me that the man ejaculates outside the vagina.” (FGD1)*

However, other women feared that it is easy for a woman to become pregnant if they relied on withdraw method and the man forget to ejaculate outside the vagina:

*“This method is not reliable because sometimes the man cannot be able to withdraw and ejaculate outside the vagina and in so doing the woman can become pregnant” (FGD 002)*

Additionally, some women mentioned traditional herbs, especially medicated strings often prescribed by traditional healers, which some women are instructed to tie around the waist to prevent pregnancy. The majority of them explained that traditional healers counsel women that they can stay up to 6–7 years without becoming pregnant as long as they wear them all the time often prescribe medicated strings:

*“There are some women who are given strings by herbalists, and they tell them that they can only become pregnant again after the string has been broken. So the string stays for six to seven years (magically protects the woman) So at that time the string breaks on its own but if it does not break it means the woman will no longer become pregnant again.” (IDI 035)*

Of those who mentioned natural family planning methods, a few expressed misinformation about them. For example, they said that not having frequent sexual intercourse is one method for natural family planning among couples:

*“A couple is not supposed to have sex frequently. That is one way of family planning for those couples who cannot use injection.” (IDI 030)*

Interestingly, the majority of women were in favour of modern family planning methods compared to natural family planning methods. This preference was based on their perception that women who normally use natural family planning methods, especially withdrawal, need to monitor their menstrual cycle each month to effectively prevent unwanted pregnancies, as opposed to using modern family planning methods. Their worry was that a woman could easily become pregnant if she does not monitor her menstrual cycle properly or if she has abnormal menstrual cycle as expressed in the quote below:

“*With natural methods, the woman does not use any medication at all, but just needs to monitor her menstrual cycle.” (IDI 016)*

#### Conflicts about the decision-making for family planning use following a poor obstetric outcome

Three groups of individuals were identified from both the IDIs and FGDs as influencing the decision making for family planning use among women following poor obstetric outcome. These were: (A) Role of the male partner, (B) Role of the woman, and (C) Role of family members,

##### Role of the male partner

In this study, the majority of women considered the issue of family planning as a family issue, which needs to be discussed between a husband and wife. They were worried especially about needing men’s support, not just for pregnancy, but also for any complications that might arise from using a family planning method:

*“Just because in a family, just as when we were coming here [meaning to attend FGDs], we told them that we are going to the hospital. So in the same way when you want to go for family planning you tell your husband that “I want to go for family planning on this day.” If he agrees to what you say he will say “Yes, you are supposed to go”. If he does not agree he will say “Why do you want to go there?” Those are your plans, and I am not part of the decision.” (FGD 003)*

Eventually, most women cited that it is important to always involve the male partner when making decisions to use family planning methods and what method to use, as opposed to other people in the family, mainly to avoid conflicts which may arise if the man realises that the wife was using family planning methods covertly. This belief could be influenced by the fact that traditionally, men are considered as the heads of households and decision-makers in all issues in their respective households, including family planning and the number of children they want to have:

*“In this case, it means they agreed, and there can be no conflict because even if a woman can use female condoms, the man can know because after sex you have to remove it. But there are some women who can go for injection without the man knowing. That can cause some conflicts in the family.” (IDI 015)*

As a result, we found that the majority of women discussed with their partners before they started using any FP methods and they were using the method which they agreed with their partner:

*“We discussed to use injection because I can stay for two months without having it and I cannon become pregnant but if I was to use condoms, sometimes my husband becomes tired and can refuse to use them. So it cannot be safe and I can become pregnant when we did not plan.” (FGD-001)*

Of great important, others reported to have gone to the clinic together with their partners for FP counselling and be able to have same information. They felt that this can help the man to develop trust on the wife:

*“We discussed that we should wait for some years before having another child and in the interim we should go for family planning and we went together for counselling before we got the services.” (FGD 003)*

##### Role of the woman

Even though the majority of women interviewed in this study considered male involvement paramount, a few women, especially those with no living child with their current partner and those who were experiencing marital problems, disagreed that men should always make the decision about family planning use. They expressed the sense that they were often the ones most aware of their overall well-being and the problems associated with frequent pregnancies, and therefore, were able to make the decision on their own whether to use contraceptives or not. One of them said:

*“We as women, we don’t need to keep quiet when it comes to childbearing issues. We don’t need to just listen and implement what the man tells us. We need to rise and tell the men the truth about our experiences, feelings and opinions regarding childbirth. He should understand that we are the ones who suffer childbirth, and we have the right to tell him how long we want to wait before having the next child.” (FGD 001)*

Some women who perceived that men should not make decisions for them, pointing out that most men discourage their women not to use family planning methods especially when the child did not live instead of encourage them.

*“If the husband is not happy that the woman gave birth to a child that did not live he can tell her not to go for family planning so that she should have another baby sooner. He would want her to go for family planning after she has a baby in her hands”. (IDI 031)*

They further considered some of the men as not being considerate about the health of the woman and just wanting them to have too many children and eventually leave them. One of them suggested covert use of FP methods that cannot be easily discovered by the husband, or use the method that can be easily reversible when they need another child so that she can protect her marriage:

*“My husband did not agree that I should be on family planning method because he wants me to become pregnant again soon but I don’t know. (…) after using the method for sometime I will stop and become pregnant as he wishes. (…) I know he will be worried but I will tell him that pregnancy is given by God so I cannot force it to happen to me.” ( FGD-004)*

However, the findings show that it was difficult for women to make decisions on their own whether to use FP methods or not especially those who are newly married or do not have a live child with the new husband despite having other children from previous marriages. They feared losing their marriages if they don’t have a child:

*“So when this happened, he suggested that I should have permanent contraception but I still want to please him. The Problem is that I have just been married to this man for one year now and I really want to have two children with him; a boy and a girl.” FGD, 002)*

##### Role of other family members

Apart from the husband, the majority of the women mentioned their friends and other family planning members, especially their mother, to have influenced their decision to start using family planning methods. This is mainly because family members, especially mothers, often escort and support pregnant women to the hospital to give birth, and they are indirectly affected with the woman’s experience during labour and delivery and the birth outcome:

*“Only my mother knows because she is the one who encouraged me to start using family planning methods.” (FGD 001)*

Others women expressed that family members encouraged them to use FP methods due to the number of children they have and also to prevent the problems from recurring One woman had this to share:

*They say it is a good decision because they have seen how I have suffered to take care of the eight children that I have. They are the ones who told me that I am supposed to go for injection before I go for permanent contraception (IDI 021)*

On the other hand, the findings show that others women are discouraged by their relatives not to use family planning methods without any living child. Most families in Malawi value to have children:

*“They tell me that if I continue using injection I will end up having no child because it affects fertility.” (FGD 004)*

Eventually, women in this study expressed a concern about the conflict they faced in deciding whose advice to follow, either to listen to other people’s concerns about their health or continue having more children and keep their marriage intact:

*“Like me, my parents told me not to give birth again. She said ‘you should go for permanent contraception. Anaemia and blood transfusion are risks.’ Initially, I agreed with their suggestions, but my husband would still want us to have another child.” FGD 002*

#### Attitudes towards family planning use after a poor obstetric outcome

##### Difference between those with and without another living child

Some women in this study were against the idea that women with no living child should use family planning methods. Their main concern was that it might not be acceptable in the community and even by family members to be seen using family planning methods without having a live child. They further pointed out that a woman with no living child expected to become pregnant again within a short period of time:

*“Because she doesn’t have a child and she is supposed to become pregnant again within a shorter period of time” (IDI 014)*

On the other hand, we found that some women were against the idea that women with no living child should not use family planning methods. Their argument was that each woman needs enough time to rest after giving birth despite that she does not have a live child:

*“The woman with a live baby may use family planning to avoid pregnancy because she wants to have good spacing between children and the woman who gave birth to a child that did not live may want to avoid pregnancy because of health problems like maybe if she was transfused blood, she may want to stay for a longer period of time so that the transfused blood mixes well in her blood” (IDI 036)*

One of them commented that the labour process does not differ whether with a live a child that did not live:

*“The process of giving birth is the same and the woman can choose to take injection, pills or Norplant” (IDI 030)*

We further found that the women with no living child were more likely to not use any family planning methods following a poor obstetric outcome, when compared women with at least one living child. For these women, their decision not to use family planning methods was mainly influenced by both their own desire and their partner’s desire to have a living child.

*Interviewer: “Why are you not using family planning methods?”*

*Respondent: “Because the child that did not live was our firstborn and we have agreed with my husband not to use any family planning methods so that I can become pregnant again and replace the child that we lost.” (IDI 15)*

##### Belief that women with a poor obstetric outcome should use different methods

The women in this study expressed different views about whether women with a poor obstetric outcome should use different family planning methods than women with a live child. Some felt that all women should be counselled and allowed to choose any family planning method which they felt comfortable to use, regardless of the delivery outcome:

*Family planning methods are the same for all women. A woman who has a live baby can use the same method that you (the woman that has a baby that did not live can use). The purpose is the same, there is no difference. (IDI 016)*

However, the majority felt that those who had a poor obstetric outcome should use different methods. They felt that women who delivered a living child should use long-acting contraceptives such as the IUD or implant so that they could wait for a longer period of time (at least three to 5 years) and have enough time to care for their baby before they became pregnant again. In contrast, the majority felt that those whose child is not alive should use short-acting methods or stay without any method so that the woman could easily become pregnant again if she wants to:

*“The woman with a live baby can use a five-year implant, while you (the woman who had a stillbirth or neonatal death) wants to use injection so that you can wait for some time, but not up to five years.” (IDI 003)*

Though many women felt that DMPA was good for those with no living child, others thought it was not a good method because they were concerned that it could take long time for a woman to conceive after she stops using it:

*“They said that if I continue using the injection I will end up having no child because it affects fertility of the woman.” (FGD 004)*

The other reason given by some for using different family planning methods was their knowledge that some family planning methods are contra-indicated in breastfeeding mothers:

*“Some of the family planning methods may not be good for women who are breastfeeding in case they may dry up the breast milk.” (IDI 015)*

##### Belief that women with a poor obstetric method can use any method

In contrast, others felt that women should be allowed to use same family planning methods regardless of the birth outcome. They explained that health providers should give all women same information about available family planning methods so that they can make an informed choice of the method they are comfortable to use:

*“It is up to you to decide whether you want to use family planning methods or not. If you decide to use family planning methods, you can choose the same methods that any woman can use.” (FGD 004)*

*“Any woman who uses family planning methods wants to prevent pregnancy, so the methods cannot be different because the purpose is the same (to prevent pregnancy).” (IDI 045)*

#### Family planning practices of participants following a poor obstetric outcome

All women in this study reported to have been encouraged to use family planning methods despite their recent poor obstetric outcome. Among women who reported using modern contraception in this study, the DMPA injectable was the most commonly reported family planning method used. They opted for this method as opposed to a daily pill because they could get it every 3 months.

*“I like injection because I was getting it every three months.” (FGD 002)*

While some women expressed worries about changes in their menstrual pattern or not having their menses at all while using DMPA, others reported that it made it easier for them to have sex with their husbands any time they wanted to do:

*“We choose this method (DMPA) because when I am using injection I don’t have menses, and this means that I can have sex with my husband anytime I want, unlike when I was using the pills because sometimes I could forget to take them.” (FGD 001)*

Although the majority of women were in favour of DMPA, others thought that oral contraceptives were the best family planning method. When asked why they considered oral contraceptives as the best family planning method, most interviewees generally stated that it is necessary for a woman to have menses monthly even if it requires that they have to take the pills every day:

*“I chose pills because unlike injection, whereby you stop having menses, with pills you do have your normal menses without any disturbance. So, some people said that you don’t feel good as a woman when you are not having menses, but pills are good because they don’t disturb your menstrual cycle.” (FGD 001)*

Interestingly, some of them considered condoms as the best contraceptive to prevent pregnancy, as well as sexually transmitted infections, including HIV. In contrast, others expressed concerns that with condoms, the woman could easily get pregnant because the husband may sometimes refuse to use them as they get tired or they feel that condoms reduce sexual pleasure, and they end up having unprotected sex. One of them explained:

*“If I was using condoms, sometimes my husband becomes tired and can refuse using them. So it cannot be safe, and I can easily become pregnant when we did not plan.” (FGD 001)*

Furthermore, some had the belief that sex with condoms is not pleasurable. Finally, others feared that if they brought condoms and asked the husband to use them, it would bring some doubts and problems between them, and they feared that the husband may think that she is suggesting that he is being promiscuous or he has a problem, such as a sexually transmitted infection. In such cases, the husband may think that their partner does not trust them anymore. One of them put it in this way:

*“Your trusted husband cannot accept to use condoms. He can refuse and say ‘It means you don’t trust me or else you think I have a problem, or else there is something that you do behind my back.’” (FGD 001*)

#### Barriers to modern family planning use following a poor obstetric outcome

##### Concern about side effects

We found that both side effects and myths/misconceptions about family planning were a barrier to modern contraceptive use among women who experienced poor obstetric outcome. Many respondents who had used modern contraception in the past mentioned real side effects as the main factors that influenced their decision not to use or discontinue using modern family planning methods. Some of the side effects cited included feeling weak, abnormal bleeding, general body pains, dizziness, nausea, constipation and lack of sexual desire:

*“I tried injection, but it was causing me to have constipation, so I had to change to pills. I eventually started using condoms because the pills were making me to feel nauseous. Condoms did not give me any problem*.” (*FGD 003)*

*“When I had just had the Norplant inserted, I had prolonged and heavy menses for one full month. When I went to the hospital, I was told that any family planning method had side effects that stops gradually. I was given treatment, but it did not stop immediately. Other women encouraged me just to remove it.” (IDI 036)*

On the other hand, some reported to have heard about the side effects from their peers, based on their first-hand experiences:

*“I heard from some of my friends that pills sometimes may cause side effects on the woman. Some of them who were saying these things have previously experienced side effects.” (IDI 015)*

The interviews also provided evidence that some women who are HIV positive decided to stop using DMPA because they were concerned about her HIV status and the side effects they were having after using DMPA. They often related the side effects experienced with major illnesses, especially cancer, which is common among HIV positive individuals:

*“You know with my HIV status, if I miss my messes, I may think of a different problem rather than the effects of the injection. I did not want to be psychologically affected because sometimes it was happening that I was feeling as if I have cancer in my stomach.” (IDI 039)*

##### Misconceptions about family planning methods

The most extreme concern cited by women in both the focus groups discussions and in-depth interviews was about prolonged use of pills and not dissolving properly; they feared that this could cause tumours and cancers, which could eventually affect their health. This belief is a result of myths/misconceptions within the community. One of them commented:

*“People talk a lot about the effects of pills. They say that they can cause an abscess in the uterus if taken for a longer period of time and if not treated properly it can cause cancer.” (FGD 004)*

In addition, some women revealed that other people in their communities related their problems experienced during labor and delivery and poor obstetric outcome to prolonged use of modern family planning methods, especially DMPA. These people believed that the medication contained in DMPA takes time to dissolve, which could affect the fetus when someone is pregnant or labor progresses:

*“They say the drug in the injection takes time to dissolve in the body, and when you are pregnant it affects the fetus.” (IDI 046)*

*“When I became pregnant I had backache from the first month up to the last month. During labor, I also had prolonged and painful labor, as compared to the experience I had with the other children. I was referred to Bwaila where I gave birth to a child which did not live. Maybe it was because I had received five injections (DMPA) before becoming pregnant, and that’s why this happened.” (IDI 032)*

## Discussion

This study examined family planning knowledge, attitudes, and practices among women who have experienced a poor obstetric outcome in Lilongwe Malawi. With regards to knowledge, the results presented here show that the majority of the women were aware of modern family planning methods as they could name most of the different modern FP methods. Antenatal and under-five clinics were predominantly mentioned as the main source of information about modern contraceptives because they all attended antenatal visits in health facilities that also offered family planning. The high level of family planning method awareness among women in this study is consistent with what was reported in the Malawi 2015–2016 DHS data with almost 98% of all women with knowledge of a modern method [5]. This also corroborates previous findings of a qualitative study conducted in Mangochi, rural Malawi where both women and men demonstrated high levels of knowledge about modern family planning methods available in the country and findings of studies conducted in other countries like Cameroon and Nigeria that have shown high level of awareness about family planning among women [12–14].

Another key finding was that despite high levels of awareness about modern family planning methods and their difficult personal experiences during labour and delivery, it was uncommon for participants to feel empowered to make decisions on their own to start using family planning methods without consulting their partners. This finding conflicts with the goals of the International Conference on Population and Development (ICPD), which includes equality between women and men in reproductive decision making and voluntary choice in determining the number and timing of one’s children [15]. The results also showed that women who have experienced a poor obstetric outcome are faced with the conflict of whether to use FP methods to protect their health, as suggested by their relatives and friends who often escorts the woman to the hospital to give birth, or to have another child to fulfill the needs of their husbands who in most cases are absent during labour and delivery. However, most women in this study had the strong opinion that men should be involved throughout the labour process and also for them to appreciate what women go through during labour and delivery and also involve them in the decision-making process for family planning use to prevent conflicts within their families. Other studies conducted in the developing countries have identified decision-making power of the husband as the main barrier for a woman’s intention to use contraceptives [16].

These key findings illustrate the need to involve men during the postpartum period and educate both the affected woman and her husband about the importance of healthy birth spacing so that they understand both economic and health benefits of family planning use, even after a poor obstetric outcome through special counselling programmes specifically designed for these couples. Available evidence demonstrates that husband’s approval was found to be a determinant of women’s intentions to use modern family planning methods as men are the primary decision-makers among couples in sub-Saharan Africa [5, 13, 17]. However, similar to the findings of previous studies conducted in many countries in Africa, men in Malawi rarely patronize maternal and child health facilities, which are often female-dominated and therefore appear intimidating and unwelcome to men [18]. Further efforts need to be made to make men feel welcome to attend antenatal and family planning visits with their partners, with emphasis that they play an important role during and after pregnancy in supporting their partner’s health so that they can have a healthy family together.

Just as in the general population of Malawi, we found that the majority of women in our study who were using a family planning method were using the DMPA injectable [5], while a few reported use of pills or condoms. This finding also corroborates with a study conducted in Kenya which showed that women preferred the DMPA injection method [19]. In contrast, our results revealed that women with no living child were not in favour of using longer-acting methods like DMPA and suggested that women with a poor obstetric outcome, particularly those with no living child should use very short-acting methods or no method at all. This was mainly due to two different reasons. First, the women expressed the need to quickly become pregnant again and have a live child to replace the child which was not alive. This agrees with the findings from a study conducted in Ghana and from data in Malawi, where parity is associated with usage of family planning methods [5, 20]. Secondly, the women noted the fear of being stigmatized by the community members if they did not have a living child. Other studies conducted in Africa have found that in societies with high fertility, child death and stillbirth were highly associated with unregulated fertility [21].

We further found that the intention to use modern family planning methods was higher among those women with previous living children than among those with no living child at all. These findings are congruent with recent national data, which shows that contraceptive use is higher among women with at least one or two children (58%) compared with those with no living child (4%) [5]. This finding is, however, worrisome given the consequences of closely spaced births (less than 15 months) and the benefits of longer pregnancy intervals [8, 21, 22]. All women, even those with a poor obstetric outcome and no living children, should be using effective contraception for at least 15 months after delivery; given the pressure that women with no living children face to have another child quickly and the fact that they will not be protected by breastfeeding. Therefore, postpartum programs need to place special emphasis on contraceptive counselling for these women and their partners.

Lastly, similar to findings from other studies conducted in the country and elsewhere, many misconceptions and concerns regarding the side effects associated with modern contraceptive methods were noted as reasons for failure to use family planning methods [5, 23–25]. Others mentioned these due to lack of proper information on how these modern methods work, e.g., thinking pills accumulate in the stomach and cause cancer. Therefore, measures must be taken to give women more information about the benefits and side effects of all modern family planning methods and allow them to make informed decisions about the method they want.

## Conclusion

Women who have experienced a poor obstetric outcome are hesitant to use family planning methods, despite being aware of them and the available evidence about the increased risks for perinatal mortality with short inter-delivery. Generally, our findings illustrate that family planning use, especially following a poor obstetric outcome, is a complex issue influenced by the woman herself, her immediate social circle, particularly the male partner, and the community in which she lives. In conclusion, our findings highlights the importance of involvement of men and significant others in family planning decision-making during the postpartum period is critical as they are often the decision makers for reproductive health issues. Involving men could help to increase awareness among men on the importance of postpartum family planning use and increase its acceptability among women in Malawi, regardless of the birth outcome. Future studies should look at better ways to involve men following bad obstetric outcome that may increase understanding of the importance of FP use even if the child did not live.

## Abbreviations

DMPA: Depo Medroxyprogesterone Acetate injectable
FGD: Focus group discussion
ICPD: International Conference on Population and Development
IDI: In-depth interview
IUD: The copper Intrauterine Device
UNC: University of North Carolina
